# Causal relationship between epigenetic markers and type 2 diabetes in West African populations: a Mendelian randomisation analysis

**DOI:** 10.1007/s00125-026-06716-3

**Published:** 2026-04-24

**Authors:** Karlijn A. C. Meeks, Eva L. van der Linden, Amy R. Bentley, Ayo P. Doumatey, Peter Henneman, Nora Franceschini, Themistocles L. Assimes, Felix P. Chilunga, Charles F. Hayfron-Benjamin, Ellis Owusu-Dabo, Guanjie Chen, Charles Agyemang, Adebowale A. Adeyemo, Charles N. Rotimi

**Affiliations:** 1 Center for Research on Genomics and Global Health, National Human Genome Research Institute, National Institutes of Health, Bethesda, MD USA; 2 Division of Endocrinology, Diabetes and Nutrition, Department of Medicine, University of Maryland School of Medicine, Baltimore, MD USA; 3 Department of Epidemiology and Public Health, University of Maryland School of Medicine, Baltimore, MD USA; 4 Department of Public and Occupational Health, Amsterdam UMC, University of Amsterdam, Amsterdam Public Health Research Institute, Amsterdam, the Netherlands; 5 Department of Human Genetics, Epigenetics, Reproduction & Development, Amsterdam UMC, University of Amsterdam, Amsterdam, the Netherlands; 6 Department of Epidemiology, University of North Carolina, Chapel Hill, NC USA; 7 Department of Genetics, University of North Carolina, Chapel Hill, NC USA; 8 Department of Medicine, Stanford University School of Medicine, Stanford, CA USA; 9 VA Palo Alto Healthcare System, Palo Alto, CA USA; 10 Department of Physiology, University of Ghana Medical School, Accra, Ghana; 11 Department of Global and International Health, School of Public Health, Kwame Nkrumah University of Science and Technology, Kumasi, Ghana; 12 Department of Medicine, The Johns Hopkins University School of Medicine, Baltimore, MD USA

**Keywords:** DNA methylation, EWAS, Mendelian randomisation, Type 2 diabetes, West Africans

## Abstract

**Aims/hypothesis:**

Evidence for a causal role of DNA methylation sites (CpGs) in type 2 diabetes and glycaemic traits is limited due to the cross-sectional nature of many epigenome-wide association studies (EWAS). In addition, epigenetic studies in West African populations are particularly sparse, despite the high and rising burden of type 2 diabetes in these populations. Hence, we aimed to identify CpGs causally associated with type 2 diabetes among West Africans by leveraging Mendelian randomisation (MR) analysis and longitudinal data.

**Methods:**

We used the Illumina EPIC DNA methylation array to profile the methylation of DNA extracted from white blood cells collected from 879 Ghanaian individuals (the Research on Obesity and Diabetes among African Migrants [RODAM] study) and 332 Nigerian individuals (the Africa America Diabetes Mellitus [AADM] study) who were not on glucose-lowering medication. We carried forwards CpGs identified in EWAS for type 2 diabetes and meta-analysed EWAS for HbA_1c_ and homeostatic model assessment estimates of insulin sensitivity (HOMA-S) as exposures to two-sample MR analysis. Independent *cis* methylation quantitative trait loci (meQTLs) were calculated using methylation data from blood and primary hepatocytes and subsequently used as instrumental variables (SNP–exposure associations). Genome-wide association analyses for type 2 diabetes on 4120 participants from the AADM study were used to derive the SNP–outcome associations. Longitudinal trait data (*n*=138) and RNA-seq data (*n*=77 blood, 49 adipose, 55 muscle) available for a subset of Nigerians were used for follow-up analyses.

**Results:**

We identified 28 CpGs associated with type 2 diabetes, 26 with HbA_1c_ and three with HOMA-S (total CpGs: 57), of which 49 had meQTLs in blood (AADM study data) and four had meQTLs in primary hepatocytes from African American individuals. MR analysis provided evidence for causality for cg00036588 and cg16759041 in type 2 diabetes using blood and hepatocyte meQTLs, respectively. Longitudinal analyses showed an association between baseline methylation of these CpGs with HbA_1c_ at follow-up. RNA-seq data revealed a *cis* correlation of cg00036588 with *FAM83C* (false discovery rate [FDR]=3.3 × 10^–4^) and *EIF6* (FDR=0.13) in skeletal muscle.

**Conclusions/interpretation:**

Our study identified two epigenetic markers as likely to be causal for type 2 diabetes in West African populations. In addition to enhancing our understanding of disease mechanisms, these CpGs with evidence of causal associations could be prioritised as potential biomarkers for early detection of disease or as drug development targets.

**Graphical Abstract:**

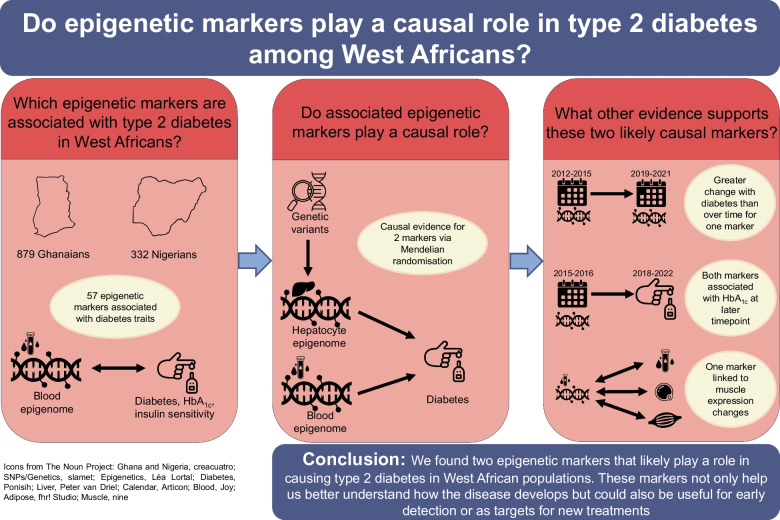

**Supplementary Information:**

The online version contains peer-reviewed but unedited supplementary material available at 10.1007/s00125-026-06716-3.


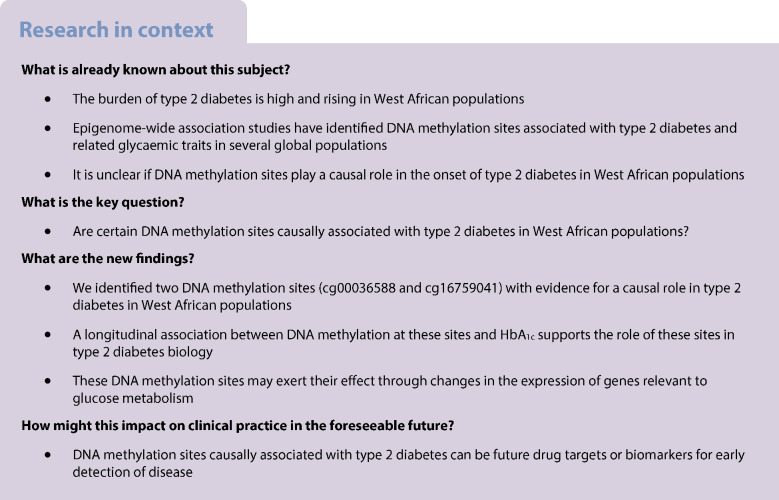


## Introduction

Type 2 diabetes is among the world’s biggest health challenges, affecting nearly 10% of the global population. This prevalence has plateaued in most high-income countries, while low- and middle-income countries have reported the highest incidence rates [1]. The number of adults with type 2 diabetes in sub-Saharan Africa is predicted to increase by 134% between 2021 and 2045 [2]. Sub-Saharan Africans residing in high-income countries have been found to be disproportionally affected by type 2 diabetes compared with their European-ancestry compatriots [3]. Exploring the unique mechanistic pathways leading to type 2 diabetes in sub-Saharan African populations could uncover targets for intervention in these individuals and beyond.

Epigenetics is one of the key mechanisms in the interplay between the genetic, environmental and lifestyle risk factors for type 2 diabetes [4], with DNA methylation being the most extensively studied epigenetic process [5]. Epigenome-wide association studies (EWAS) have identified DNA methylation sites for type 2 diabetes across 130 genes [6], but these EWAS have been predominantly performed in populations of European ancestry, and studies in sub-Saharan African individuals are particularly scarce [7]. In the only EWAS for type 2 diabetes performed in a sub-Saharan African population thus far, four DNA methylation type 2 diabetes sites were identified, of which one (cg07988171, annotated to *TPM4*) was novel and potentially population specific [8]. There is a need for more EWAS among sub-Saharan African populations, given the high genetic diversity of African-ancestry populations and the wide range of environmental exposures. These unique characteristics provide an opportunity to advance our understanding of the role of DNA methylation in type 2 diabetes [9]. Additionally, better characterisation of DNA methylation changes is important, because epigenetic markers are vulnerable to confounding and reverse causation. Identifying causal DNA methylation sites in type 2 diabetes will aid in the selection of such sites as early biomarkers to detect type 2 diabetes or in identifying targets for treatments based on epigenetic mechanisms [10].

Mendelian randomisation (MR) is an approach that can, under specific assumptions, be used to infer causality, with particular application to observational studies. MR analyses use genetic variants as proxies of exposure (instrumental variables) to establish whether an exposure is causally associated with an outcome. The random inheritance of genetic variants is at the core of this technique, as these randomly inherited variants are not affected by confounding or reverse causation. The application of the MR method to DNA methylation studies is still relatively new. MR can be applied to DNA methylation when independent methylation quantitative trait loci (meQTLs) are selected as the instrumental variables. We aimed to identify DNA methylation sites associated with type 2 diabetes and glycaemic traits in West African populations and to assess causality in the association between these sites and type 2 diabetes using meQTL-based MR.

## Methods

### Data and populations

**Fig. 1:**
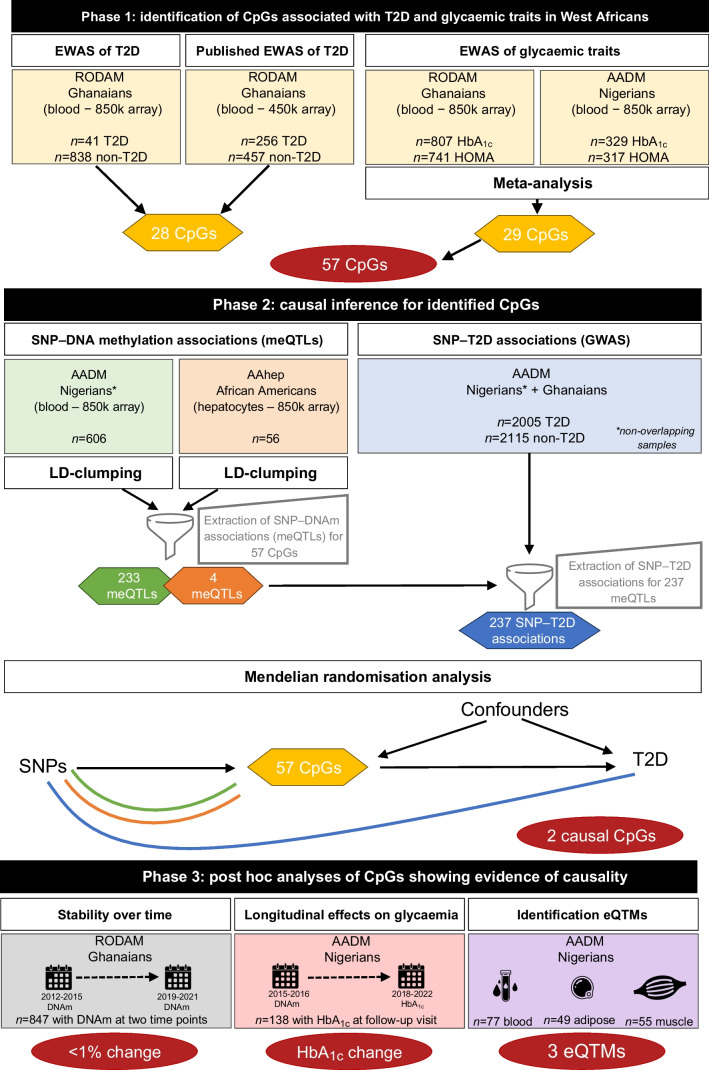
Overview of datasets (coloured boxes), analyses (white boxes) and results (red ovals). Icons by creators from thenounproject.com: filter icon by Garis Tanam, blood icon by Joy, adipose icon by Fhrustrator, muscle icon by Nine, calendar by Articon. DNAm, DNA methylation; T2D, type 2 diabetes

We used data from two West African cohorts, namely the Research on Obesity and Diabetes among African Migrants (RODAM) study and the Africa America Diabetes Mellitus (AADM) study. An overview of the data, populations and analyses can be found in Fig. 1.

#### The RODAM study

The RODAM study aims to understand the reasons for the high prevalence of obesity and type 2 diabetes among the sub-Saharan African diaspora. Between 2012 and 2015, Ghanaian men and women residing in rural Ghana, urban Ghana, London, Berlin and Amsterdam were recruited. For the present analyses, only Ghanaians in rural Ghana, urban Ghana and Amsterdam were included. Recruitment procedures have been described in detail previously [11, 12]. In brief, two cities (Kumasi and Obuasi) and 15 villages in the Ashanti region of Ghana were used as the sampling frame for urban and rural Ghana sites, respectively. Participants were randomly drawn from a list of 30 enumeration areas based on the 2010 census. Ghanaians living in Amsterdam were considered Ghanaian if they were born in Ghana with at least one parent being born in Ghana (first generation) or if they were born in the Netherlands but both of their parents were born in Ghana (second generation). Eligible participants (Ghanaian ethnicity and age >18 years) were randomly selected from municipal registers. Random sampling from registers facilitated representativeness. However, individuals not captured in these registers, such as non-documented migrants, may differ in terms of demographic and socioeconomic characteristics. Overall, the RODAM study sample is broadly representative of the target populations in terms of age and sex distribution. Follow-up data collection was performed between 2019 and 2021 for participants who completed baseline data collection and resided in rural Ghana, urban Ghana or Amsterdam. RODAM study ethical approval was obtained from the respective ethics committees in Ghana (School of Medical Sciences/Komfo Anokye Teaching Hospital Committee on Human Research, Publication & Ethical Review Board) and the Netherlands (Institutional Review Board of the AMC, University of Amsterdam) before data collection began in each country. All participants gave written informed consent, including permission for long-term storage and future use of their data and biospecimens for health-related research, including studies of gene–disease relationships and epigenetic markers. Participants could opt out of specific analyses if they wished.

#### The AADM study

The AADM study enrolled individuals with type 2 diabetes and individuals without type 2 diabetes (control participants) from university medical centres in West Africa and in East Africa [13–15]. The overarching aim of the AADM study is to map type 2 diabetes susceptibility genes in African populations [14, 15]. Recruitment procedures have been described in detail previously [13–15]. In brief, data have been collected across four phases, beginning in 1997. Eligible participants were aged 25 years and over, with or without type 2 diabetes. The study attempted to enrol equal numbers of men and women (the overall proportion of women was approximately 60%). The major ethnolinguistic groups in the overall study were Yoruba, Ibo, Akan, Ga-Adangbe, Kalenjin, Kikuya and Luhya, reflecting the major ethnolinguistic groups at the study sites. Participants were recruited from medical centres in urban settings and surrounding communities. Therefore, the sample was representative of individuals receiving care in urban communities, who may differ in terms of socioeconomic status, cardiometabolic factors and ethnicity from rural communities. The most recent phase, a longitudinal study including epigenetics profiling, collected baseline data between 2015 and 2016. The present analyses only included West African participants who were recruited from three cities in Nigeria (Ibadan, Enugu and Lagos) and two cities in Ghana (Accra and Kumasi). West African ancestry was confirmed genetically. Ethical approval for the AADM study was obtained from the NIH and from the respective ethical committees in Ghana and Nigeria. During the consent process, all participants were informed that their data and samples would be stored indefinitely and used for unspecified future research on diabetes and other diseases. All participants provided written, informed consent.

#### Community engagement and capacity building

Extensive community engagement was carried out prior to and during the original sampling for both the AADM and the RODAM studies. In parallel, both studies prioritised capacity building by supporting the training of local investigators, PhD students, postdoctoral fellows and nurses through opportunities for data analysis and authorship. Notably, several investigators involved in these studies have since become independent scientists at both national and global levels.

### Phenotypic measurements

Demographics and health-related behaviours were assessed in both the RODAM and the AADM studies, assessed through interviews or self-administered questionnaires. Sex was determined through self-report in both studies and confirmed genetically in the AADM study. Current smokers were those who responded affirmatively to the question ‘Do you smoke?’ (RODAM) or reported smoking regularly for the past 1 year (AADM). Height (m) and weight (kg) were measured by trained staff during physical exams and BMI was subsequently calculated as kg divided by m^2^. Fasting serum and plasma samples were obtained by trained personnel. In the RODAM study, fasting glucose was measured using the enzymatic hexokinase method and HbA_1c_ was measured using high-performance liquid chromatography (Tosoh G8 HPLC analyser, Tosoh Bioscience, Tokyo, Japan). In the AADM study, fasting glucose concentration was measured using the enzymatic reference method with hexokinase on the Roche Modular-E autoanalyzer (Roche Diagnostics, Indianapolis, IN, USA). HbA_1c_ was measured in the AADM study for a subset of 650 individuals recruited in Nigeria using the latex agglutination inhibition immunoassay on DCA Vantage (Siemens Medical Solutions USA, Inc, Malvern, PA, USA). Insulin concentrations were measured in the RODAM study using the Mercodia ELISA kit (Mercodia, Uppsala, Sweden) and in the AADM study by electrochemiluminescence immunoassay (ECLIA) on Roche Modular-E (Roche Diagnostics, Indianapolis, IN, USA). Type 2 diabetes was defined as a fasting plasma glucose of ≥7.0 mmol/l (126 mg/dL), self-reported type 2 diabetes following physician diagnosis or the use of glucose-lowering medication. We used the updated homeostatic model assessment (HOMA) from the University of Oxford to estimate insulin sensitivity (HOMA-S) and beta cell function (HOMA-B) based on measured insulin and fasting glucose concentrations. The updated HOMA model is a computer model that derives HOMA-S and HOMA-B as percentages of a normal reference population rather than linear approximations (available from www.dtu.ox.ac.uk/homacalculator/).

### DNA methylation data

We used DNA methylation data available for a subset of 905 RODAM study samples and 615 AADM study samples. The main RODAM subset consisted of Ghanaian individuals recruited at baseline in rural Ghana, urban Ghana and Amsterdam. For 847 RODAM samples, DNA methylation was profiled at follow-up as well. The AADM subset consisted of Nigerian individuals recruited from a single site, namely Ibadan, Nigeria. DNA methylation profiling was performed using the Illumina Infinium methylation EPIC BeadChip array on DNA from whole blood samples by Erasmus MC Core Facility for the RODAM samples and on DNA from peripheral blood mononuclear cells (buffy coat) by the University of Colorado for the AADM samples. This microarray provides coverage to interrogate over 850,000 methylation sites across the genome. Bisulfite conversion of extracted DNA was done using sodium bisulfite to convert unmethylated cytosine to uracil and the bisulfite-converted DNA was subsequently amplified and hybridised to the BeadChip. For the RODAM study, which includes DNA methylation data at two time points, samples were randomised across 27 plates. For the AADM study, samples were randomised across seven plates, divided over two batches. Both studies included control samples on each plate to minimise potential batch effects and monitor assay performance. The quality-control procedures, including checks for batch effects, were performed on RODAM data using the *MethylAid* R/Bioconductor package [16] (version 1.30.0) and revealed 27 outliers and 15 sex anomalies for exclusion. Quality control using the *Sesame* R/Bioconductor package (version 1.26.0) [17] on AADM samples revealed one sample having failed the bisulfite conversion, three sex anomalies and six outliers based on principal component analyses and evaluation of mean intensities. The raw DNA methylation data for samples passing quality-control procedures were normalised using the preprocessFunnorm function as implemented in the *Minfi* R/Bioconductor package (version 1.48.0) [18]. *M* values were calculated as the log2 ratio of the intensities of methylated probes vs unmethylated probes. All analyses were based on *M* values rather than beta values, as *M* values are deemed more statistically valid [19]. We removed all CpG sites annotated to chromosomes X or Y, as well as cross-reactive probes. Cell types were estimated using the method developed by Houseman et al [20], as implemented in the *FlowSorted.Blood.EPIC* R/Bioconductor package (version 2.12.0) [21].

### Genotyping data

Genotyping data were available only for the AADM study participants. Genotyping and imputation procedures have been described in detail previously [13]. In brief, participants’ samples were genotyped using the Affymetrix Axiom PANAFR SNP array or Illumina’s Multi-Ethnic Global Array. All samples had a sample-level genotype call rate of at least 0.95 after quality control. The SNP datasets were filtered for missingness per marker (>5%), minor allele frequency (<1%) and deviations from the Hardy–Weinberg equilibrium (*p* value ≤1×10^−6^) and imputed using the Trans-Omics for Precision Medicine (TOPMed) mixed population imputation panel.

### RNA-seq data

RNA-seq was carried out by the NIH Intramural Sequencing Center (NISC) on 77 blood samples, 49 white adipose tissue samples and 55 skeletal muscle tissue samples from AADM participants who also had DNA methylation data. Total RNA was extracted using a miRNeasy Mini kit (217004, Qiagen) in randomised batches. Poly(A)-selected, stranded mRNA libraries were then prepared using Illumina TruSeq Stranded mRNA sample preparation kits and sequenced on a HiSeq 2500 with version 4 chemistry, targeting a minimum of 45 million 126-base paired-end reads per sample, with an average of approximately 87.9 million reads per sample. Reads were aligned to the GRCh38 genome reference downloaded from the UCSC Genome Browser using RSEM v 1.3.3 and BOWTIE2 2.5.3 with standard parameters [22, 23]. Gene expression levels were subsequently calculated using RSEM expressed as transcripts per million.

### Data analyses: phase 1 – identification of DNA methylation markers

#### EWAS for type 2 diabetes

To identify DNA methylation loci significantly associated with type 2 diabetes, we performed EWAS for type 2 diabetes in Ghanaians (Fig. 1). We fitted a linear regression model adjusted for age, sex, recruitment site, cell proportions, BMI and plate using the RODAM study data. Sex was included as a covariate in all statistical models to account for potential biological differences. The quantile–quantile (Q–Q) plot can be found in electronic supplementary material (ESM) Fig. 1a. Given the substantial effect of glucose-lowering medication on DNA methylation [24], we excluded all samples of participants who reported using these medications from EWAS analyses. Hence, these analyses included 41 individuals with type 2 diabetes not on glucose-lowering medication and 838 individuals without type 2 diabetes. Since AADM participants were recruited through medical centres, the cohort did not include individuals with undiagnosed type 2 diabetes, and all individuals with type 2 diabetes were receiving glucose-lowering medication. Given the large effect of these medications on DNA methylation [24], we did not perform EWAS for type 2 diabetes using this cohort. Instead, we additionally considered the four CpGs (cg19693031, cg04816311, cg00574958 and cg07988171) identified in the only previously published EWAS for type 2 diabetes in West Africans [8]. This previously published EWAS was performed on a subset of 713 RODAM samples using the Illumina 450k array and thus covered fewer CpG sites, but it was better powered for the binary type 2 diabetes outcome with 256 individuals with type 2 diabetes not on glucose-lowering medication and 457 individuals without type 2 diabetes. There was an overlap of 109 samples (36 with type 2 diabetes and 73 without type 2 diabetes) with the RODAM study EWAS reported in this manuscript.

#### EWAS for glycaemic traits

To identify additional DNA methylation sites relevant for type 2 diabetes-related glycaemic traits, we performed EWAS analyses on natural log-transformed HOMA-S, HOMA-B and HbA_1c_ using individuals not on glucose-lowering medication from both RODAM and AADM data, encompassing both individuals with undiagnosed/untreated type 2 diabetes and individuals without type 2 diabetes. Models were adjusted for age, sex, cell proportions, BMI and technical covariates for both cohorts (plate for the RODAM study; plate and plate position for the AADM study). The RODAM models were additionally adjusted for recruitment site. Analyses were conducted using complete-case analysis to handle missing data. The RODAM and AADM study summary statistics for HbA_1c_, HOMA-S and HOMA-B were subsequently meta-analysed per trait using METAL (release 25 March 2011) [25]. Q–Q plots demonstrated well-controlled inflation (ESM Fig. 1b, c, d). CpG sites identified in meta-analysis at a Benjamini and Hochberg false discovery rate (FDR) of <0.05 were considered robustly associated with type 2 diabetes and related traits and carried forwards.

As a sensitivity analysis to account for >5% missingness in HbA_1c_ and HOMA measures in the RODAM study, we repeated the EWAS using glycaemic traits imputed via multivariate imputation by chained equations in R. HbA_1c_, insulin and glucose were imputed using age, sex, study site, type 2 diabetes status, BMI, smoking status and waist circumference as predictors. HOMA-S and HOMA-B were then recalculated from the imputed insulin and glucose values. Subsequent meta-analyses with the AADM study were repeated, and results were compared with those from the complete-case analysis. For HbA_1c_, genomic inflation improved after imputation (λ=1.05 vs 1.19), whereas model fit worsened for the HOMA measures (λ=1.20 vs 0.97 for HOMA-B and 1.85 vs 1.41 for HOMA-S).

### Data analyses: phase 2 – causal inference for identified DNA methylation markers

#### SNP–exposure associations: identification of blood and hepatocyte-specific meQTLs

Identification of independent causal variants for DNA methylation sites (meQTLs) was performed genome wide, leveraging AADM study participants who had both genotyping data and DNA methylation data on white blood cells (*n*=606; *n*=280 with type 2 diabetes). White blood cells are the most studied tissue type in DNA methylation analyses. Given the tissue specificity of DNA methylation data and the well-documented lack of correlation of meQTLs across tissues, we additionally identified meQTLs using DNA methylation data from hepatocytes. Our goal was to explore whether any CpGs exhibit cross-tissue effects, potentially indicating a causal role across tissues. Hepatocytes are relevant in the pathophysiology of type 2 diabetes, as they play a central role in glucose metabolism and insulin sensitivity and are targets of some type 2 diabetes medications. Hepatocyte meQTL analysis was performed using publicly available DNA methylation and genotyping data from the Gene Expression Omnibus (GEO) database deposited with the 2019 publication by Park et al [26] (GSE124076). Specifically, DNA methylation data were obtained from GEO accession number GSE123995, which included Illumina Infinium methylation EPIC BeadChip array data from primary hepatocyte samples of 56 African American individuals with a median West African ancestry of 83%. Corresponding genotyping data generated using the Illumina Multi-Ethnic Genotyping Array for these 56 samples were retrieved from GEO accession number GSE147628. Henceforth, we refer to this dataset as ‘AAhep’. We processed these DNA methylation and genotyping data according to the same procedures as described previously for the RODAM and AADM datasets.

Both blood and hepatocyte meQTLs were identified by associating all imputed genetic variants with an imputation quality score (INFO score) ≥0.8 on normalised *M* values for DNA methylation sites passing quality control. We used an additive genetic model in a linear mixed model framework as implemented in the R package QTLtools (version 1.3.1) [27], with age, sex, type 2 diabetes medication use and estimated cell proportions as covariates for the AADM study, and age, sex and proportion West African ancestry as covariates for the AAhep data. Genetic variants overlapping DNA methylation probes were excluded from all meQTL analyses. *Cis* quantitative trait loci within a 2 Mb region around each CpG site of interest with an FDR <0.05 were carried forwards as potential instruments for MR analyses. The nearest meQTL was at a 34 bp distance from the CpG. To ensure that the instruments for the DNA methylation loci were independent, we performed a pairwise linkage disequilibrium (LD) analysis commonly known as ‘LD clumping’ using PLINK 1.9 [28] with an *r*^2^ threshold of <0.1 and using the imputed AADM and AAhep genotyping data as the LD reference for the blood and hepatocyte analyses, respectively. The SNPs with the lowest *p* value in each clump were selected as the independent instrument for that clump. It is important to note that these *p* values are presented descriptively in the results to indicate instrument choice, but are not interpreted as formal significance tests, as selection induces a different null distribution.

MR relies on three core assumptions: (1) genetic variants used as instrumental variables are robustly associated with the exposure (relevance assumption); (2) instrumental variables are not associated with confounders (independence assumption); and (3) instrumental variables affect the outcome only through the exposure and not via other pathways (exclusion restriction). To assess these first two core instrumental variable assumptions, we undertook several quality control steps. To ensure the relevance assumption, we calculated cumulative *F* statistics of all instruments per CpG site. All instruments had *F* statistics >10, so all were retained in the MR analyses. To evaluate the independence assumption, we regressed all independent instruments on age, sex, BMI, waist circumference, hip circumference and smoking status using the data from the larger AADM study. We excluded instruments with evidence of potential pleiotropy (association *p* value <0.05).

#### SNP–outcome associations: type 2 diabetes genome-wide association studies

Genome-wide quantitative linear regression analyses were performed for type 2 diabetes on 4120 AADM West African participants who were not included in the DNA methylation subset to prevent sample overlap in the MR analyses. A mixed linear model was fitted using the GENESIS R/Bioconductor package (version 2.38.0) [29] assuming an additive genetic model with adjustment for age, sex, the first three principal components, BMI and a genetic relatedness matrix. While these covariates differed from those used in the SNP–exposure models (blood and hepatocyte meQTLs), they were tailored to the characteristics of each dataset. Cell composition was relevant for blood DNA methylation data, ancestral admixture for the hepatocyte dataset in African Americans and population structure for the outcome genome-wide association study (GWAS).

#### MR analysis

The MendelianRandomization package in R (version 0.1) [30] was used to perform two-sample MR analysis using identified instruments in blood and in hepatocytes separately. Considering the MR exclusion restriction assumption, we followed the Rucker framework, which uses goodness-of-fit heterogeneity statistics to systematically decide model selection. Following this framework, we first performed MR analyses using the fixed effects inverse variance weighted (IVW) method for CpGs with multiple meQTL instruments. For CpGs with a single meQTL instrument, the Wald ratio was used. Cochran’s *Q* statistic for the IVW analyses was used as an assessment of heterogeneity. If the IVW fixed effects model had a Cochran’s *Q p* value of <0.05, a random effect IVW model was fitted. Next, Rucker’s *Q* statistic was calculated to determine if MR Egger analysis should be performed.

### Data analyses: phase 3 – post hoc analyses of DNA methylation markers showing evidence of causality

#### Stability of causal DNA methylation markers

To gain more insight into CpGs with evidence for causality, we assessed the stability of the CpGs over time. For 847 RODAM samples, DNA methylation data were available for a second time point collected between 2019 and 2021, a mean of 6.7 years after baseline (Fig. 1). We extracted the normalised betas per sample at both time points for identified CpG sites. We then calculated the mean methylation percentage for each CpG site at time points 1 and 2 as well as the difference in mean methylation between both time points.

#### Temporality of DNA methylation changes

Next, we assessed the temporality of DNA methylation changes for the CpGs with evidence for causality. In AADM, longitudinal data were available for 138 individuals (*n*=6 with type 2 diabetes but not on medication) who had DNA methylation measured at baseline and HbA_1c_ measured at a follow-up, which occurred a mean of 3.8 years after baseline. These individuals were not on glucose-lowering medication at either baseline or follow-up. HbA_1c_ levels were categorised into tertiles and the baseline methylation percentage for each CpG was plotted per HbA_1c_ tertile.

#### Correlation between expression quantitative methylations and gene expression levels

Finally, we calculated expression quantitative methylations (eQTMs) to explore correlations between causal CpGs and RNA-seq gene expression in blood, skeletal muscle and white adipose tissue. The Matrix eQTL tool (version 2.3) [31] was used for these analyses because of its flexibility in input formats (methylation *M* values instead of SNPs). Both *cis* (within 1 Mb of the CpG site) and *trans* (more than 1 Mb away from the CpG site) eQTMs were calculated genome wide, with adjustment for age, sex and type 2 diabetes medication use for each of the three tissues separately. Given the small sample size and explorative nature of these post hoc analyses, eQTMs reaching a threshold FDR of <0.2 were considered of potential relevance in addition to the standard FDR <0.05 significance threshold.

## Results

### Characteristics of the study populations

The characteristics of the West Africans from the RODAM and AADM studies included in the DNA methylation, meQTL and GWAS analyses are shown in Table 1, with Fig. 1 providing an overview of subset usage. Differences in glycaemic traits across subsets were expected due to the higher number of individuals with undiagnosed type 2 diabetes in the RODAM study and the type 2 diabetes-enriched meQTL and GWAS datasets.

**Table 1:** Participant characteristics

Characteristic	EWAS		meQTLs		GWAS
	RODAM cohort	AADM cohort	AADM cohort	AAhep cohort	AADM cohort
*N*	879	332	606	56	4120
Sex, women, *n* (%)	551 (62.7)	254 (76.5)	472 (77.9)	25 (44.6)	2474 (60.0)
Location, *n* (%)					
Rural Ghana	321 (36.5)	0 (0)	0 (0)	0 (0)	0 (0)
Urban Ghana	289 (32.9)	0 (0)	0 (0)	0 (0)	1625 (39.4)
Urban Nigeria	0 (0)	332 (100)	606 (100)	0 (0)	2495 (60.6)
Amsterdam	269 (30.6)	0 (0)	0 (0)	0 (0)	0 (0)
USA	0 (0)	0 (0)	0 (0)	56 (100)	0 (0)
Age, years, mean ± SD	46.7±11.0	55.4±12.6	57.5±11.9	39.2±19.3	49.6±14.1
Type 2 diabetes, *n* (%)	41 (4.7)	6 (1.8)	280 (46.2)	NA	2005 (48.7)
Type 2 diabetes medication use, *n* (%)	0 (0)	0 (0)	274 (45.2)	NA	1929 (46.8)
Biguanides	0 (0)	0 (0)	263 (43.4)	NA	1347 (32.7)
Non-biguanides	0 (0)	0 (0)	11 (1.8)	NA	451 (10.9)
Unknown type	0 (0)	0 (0)	0 (0)	NA	131 (3.2)
HbA_1c_, %, median (IQR)	5.4 (4.9–5.7)	5.4 (5.2–5.7)	5.8 (5.4–7.3)	NA	5.8 (5.3–7.3)
HbA_1c_, mmol/mol, median (IQR)	35.7 (30.5–39.1)	35.5 (33.3–38.8)	39.9 (35.5–56.3)	NA	39.3 (33.9–56.0)
HOMA-S, median (IQR)	134.9 (85.6–223.6)	114.3 (75.9–179.5)	98.4 (66.0–156.1)	NA	97.7 (53.1–190.8)
HOMA-B, median (IQR)	74.8 (53.0–98.6)	106.0 (79.1–138.7)	85.0 (48.3–123.4)	NA	66.7 (32.1–109.4)
BMI (kg/m^2^), mean ± SD	26.0±5.2	30.8±6.4	31.2±6.4	NA	26.4±5.7
Smoker, *n* (%)	21 (2.4)	1 (0.3)	6 (1.0)	NA	151 (3.7)
Cell counts, %, mean ± SD					
CD8+ T cells	13.7±4.8	14.6±4.5	14.6±4.6	NA	NA
CD4+ T cells	21.3±6.0	20.1±5.6	19.7±5.7	NA	NA
Natural killer cells	5.0±2.9	5.6±2.8	5.4±2.9	NA	NA
B cells	8.3±3.2	9.2±5.2	8.7±4.3	NA	NA
Monocytes	8.1±2.7	8.3±2.4	8.2±2.4	NA	NA
Neutrophils	43.2±11.8	41.5±9.8	42.8±9.9	NA	NA

NA, not applicable

### Observational associations between DNA methylation and type 2 diabetes-related traits

**Fig. 2:**
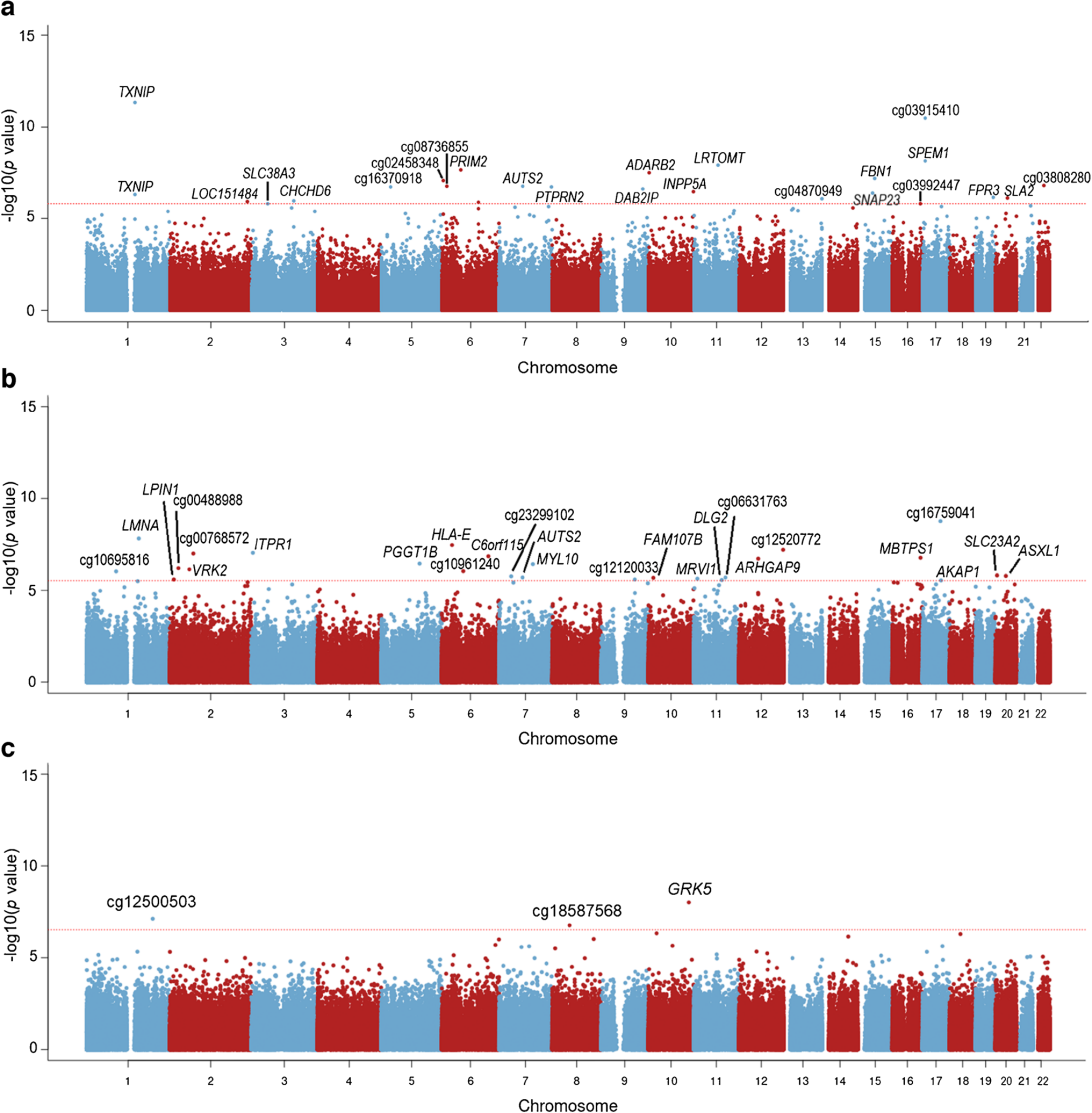
Manhattan plot of epigenome-wide *p* values for (**a**) type 2 diabetes, (**b**) HbA_1c_ and (**c**) HOMA-S. (**a**) EWAS were conducted in 879 Ghanaians for type 2 diabetes. (**b**) Meta-analysed EWAS were conducted on 806 Ghanaians and 329 Nigerians for HbA_1c_. (**c**) Meta-analysed EWAS were conducted on 741 Ghanaians and 317 Nigerians for HOMA-S. The red dotted line indicates epigenome-wide significance according to FDR multiple test correction (FDR <0.05). This FDR corresponds to a *p* value of 1.6 × 10^−6^ for type 2 diabetes (**a**), 2.9 × 10^−6^ for HbA_1c_ (**b**) and 2.5 × 10^−7^ for HOMA-S (**c**). UCSC gene annotations were derived through the Illumina human methylation EPIC array manifest. Intergenic CpGs were annotated using their cg IDs

EWAS for type 2 diabetes using the 850k RODAM data identified one overlapping CpG (*TXNIP*) with the previously published EWAS for type 2 diabetes in West Africans using the 450k array and 24 additional CpGs [8], 15 of which were not covered by the 450k array (Fig. 2a and ESM Table 1). Including the four CpGs from that previous study, a total of 28 type 2 diabetes-associated CpGs were identified. Meta-analyses of EWAS for type 2 diabetes-related traits revealed 26 CpGs associated with HbA_1c_ and three with HOMA-S (Fig. 2b, c and ESM Table 2). No genome-wide significant CpGs were detected for HOMA-B. The total of 57 identified CpGs were carried forwards as exposures to two-sample MR analysis.

The sensitivity analysis using imputed glycaemic traits identified 20 CpGs for HbA_1c_, 20 for HOMA-S and none for HOMA-B. FDR values from the imputed analysis are reported in ESM Table 2 alongside those from the complete-case analysis, allowing assessment of consistency across approaches. Additional CpGs identified only in the imputed analysis are not discussed because of the increased genomic inflation suggesting that these results should be interpreted with caution.

### Causal associations between DNA methylation and type 2 diabetes

**Fig. 3:**
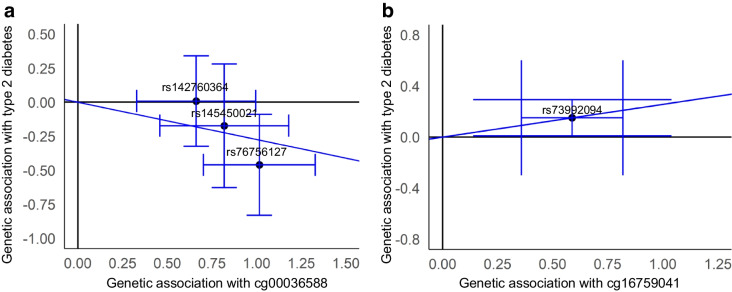
Scatter plot visualising the causal estimates on type 2 diabetes of (**a**) cg00036588 in blood and (**b**) cg16759041 in hepatocytes. The SNPs shown are the independent instruments retained after LD clumping (*r*^2^ <0.1), with (**a**) three SNPs for cg00036588 and (**b**) one SNP for cg16759041. The error bars denote the SE of the SNP–CpG (*x*-axis) and SNP–type 2 diabetes (*y*-axis) associations. The diagonal blue line indicates the causal estimate

Following LD clumping, we identified 289 independent blood *cis* meQTLs for 53 of the 57 selected CpGs corresponding to 64 genomic regions. After exclusion of meQTLs nominally associated with potential confounders, 233 *cis* meQTLs within a 2 Mb distance remained across 49 CpGs (ESM Table 3). SNP–type 2 diabetes associations are shown in ESM Table 4. Of the 49 CpGs, 36 had multiple independent SNP instruments, allowing for multi-instrument MR analyses using IVW and calculation of heterogeneity statistics. Using the MR IVW fixed effects model, cg00036588 was causally associated with type 2 diabetes at a nominally significant *p* value of 0.039 (OR=0.76) (Fig. 3a and ESM Table 5). The instrument consisted of three SNPs (rs142760364, rs145450021 and rs76756127) and the causal estimate showed no evidence for heterogeneity based on Cochran’s *Q* statistic (*p*=0.327). Two of the IVW estimates had significant heterogeneity (Cochran’s *Q*) and, therefore, were carried forwards to the IVW random effects model, following the Rucker framework. Rucker’s *Q* statistic was low to moderate for both of these CpG sites suggesting appropriate model fit (ESM Table 6). Neither of these was significant in either the IVW fixed or the random effects models.

In a complementary, less restrictive analysis including 259 *cis* meQTL instruments across 53 CpGs excluding only those nominally associated with age and/or sex, no MR associations reached *p*<0.05 using IVW fixed effects models (ESM Table 7). For cg00036588, expanding the instrument set attenuated the effect estimate and introduced significant heterogeneity. Significant heterogeneity remained with alternative modelling under the Rucker framework (Rucker’s *Q*=4.65, *p*=0.031), including MR Egger (OR=0.24, 95% CI 0.04, 1.41; *p*=0.11).

Using hepatocyte DNA methylation data, meQTLs were identified for only four CpGs (ESM Tables 8 and 9). The Wald ratio MR analyses using the four single meQTLs as instruments provided nominal evidence for one causal CpG (Fig. 3b and ESM Table 10). Using rs73992094 as the instrument, CpG site cg16759041 was associated with type 2 diabetes at an OR of 1.29 and a *p* value of 0.037. As only single-SNP instruments were available per CpG, heterogeneity statistics could not be calculated. Notably, this cg16759041 CpG was identified in the meta-analysed EWAS of glycaemic traits and remained robust across complete-case and imputed analyses (FDR=3.40 × 10^−4^ vs 8.37 × 10^−4^).

### Stability over time of causal CpG sites

**Fig. 4:**
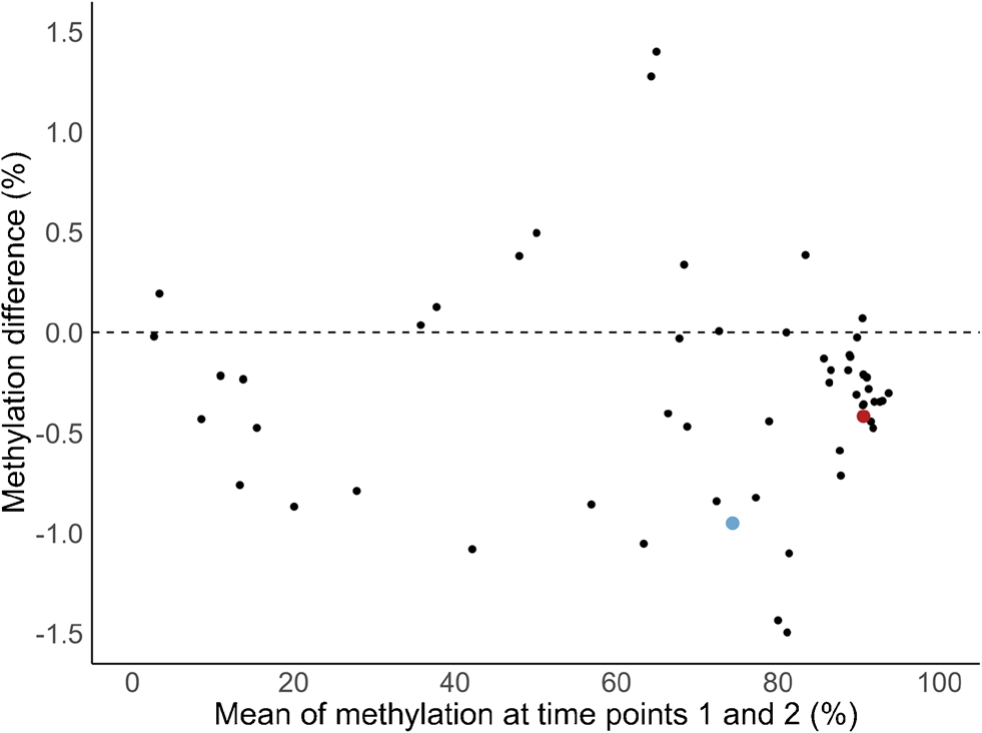
Bland Altman plot visualising the stability of the 57 CpGs between two time points about 6 years apart. CpG cg00036588 is highlighted in red and cg16759041 is highlighted in blue

Stable CpG sites, especially in blood, have the potential to serve as biomarkers. We found most of the 57 CpG sites to vary by less than 1% between two time points about 6 years apart (Fig. 4). At time point 2, for the causally associated CpGs, the mean methylation level in blood was 0.42% lower for CpG cg00036588 and 0.95% lower for cg16759041. For cg00036588, this 7-year change is modest relative to the EWAS effect size, as type 2 diabetes was associated with a 2.7% lower methylation level, suggesting that the type 2 diabetes-associated variation exceeds the variability over time. For cg16759041, EWAS meta-analysis showed that a 10 mmol/mol increase in HbA_1c_ was associated with 0.9% lower methylation, which is of a similar magnitude to the 0.95% decline observed over 7 years.

### Longitudinal effects of causal CpG sites

**Fig. 5:**
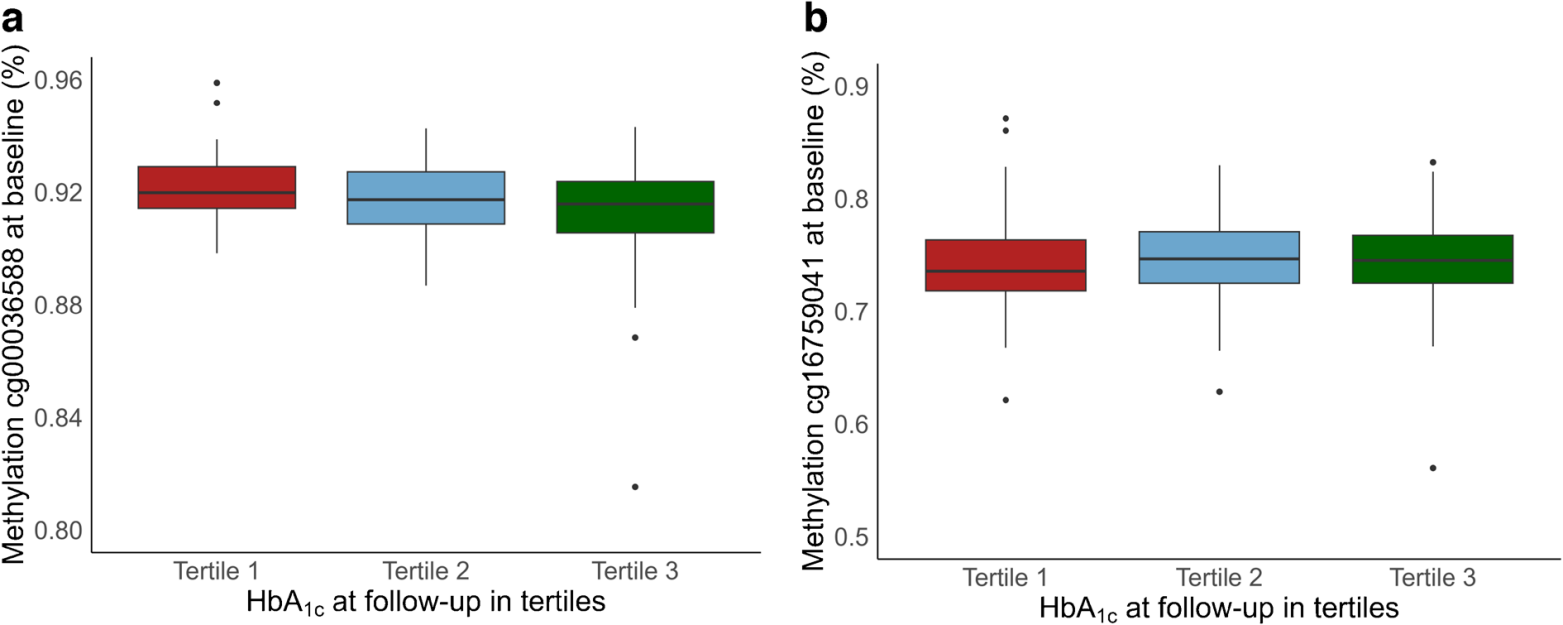
Boxplot of methylation levels for (**a**) cg00036588 and (**b**) cg16759041 at baseline by HbA_1c_ tertile at second follow-up. The centre line represents the median and the lower and upper bounds of the box represent the first and third quartiles, respectively. The whiskers extend to the minimum and maximum values within 1.5 times the IQR, with individual points representing outliers

Using longitudinal data from the AADM study, we found that the median percentage methylation at baseline for cg00036588 was higher among those with lower HbA_1c_ at second follow-up (92.0%, 91.7% and 91.6% for HbA_1c_ tertiles 1 to 3, respectively; Kruskal–Wallis χ^2^ *p* value=0.05), which is consistent with our findings from the MR analysis suggesting that higher methylation is protective of type 2 diabetes (Fig. 5a). MR analysis showed CpG cg16759041 to be causally associated with higher odds for type 2 diabetes and, accordingly, those in the middle and highest tertiles of HbA_1c_ at follow-up were found to have higher methylation at baseline for this CpG (73.6%, 74.7% and 74.5% for HbA_1c_ tertiles 1 to 3, respectively; Kruskal–Wallis χ^2^ *p* value=0.03) (Fig. 5b).

### Correlation of causal CpG sites with gene expression

**Fig. 6:**
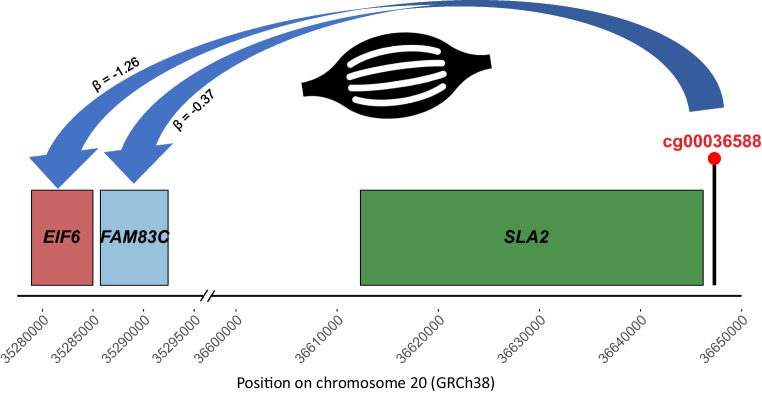
Schematic of regional eQTM effect of cg00036588 in muscle tissue. Muscle icon by Nine, from thenounproject.com

Two eQTMs were detected for cg00036588 at an FDR <0.05: the RNA gene *LOC105372599* in blood (beta=−0.045, FDR=1.38 × 10^−6^) and *FAM83C* in skeletal muscle (beta=−0.366, FDR=3.3 × 10^–4^). An additional association with *EIF6* in skeletal muscle was observed at a more relaxed threshold (beta=−1.258, FDR=0.13). Figure 6 shows the genomic locations of cg00036588 and the associated genes in muscle tissue. CpG cg00036588 is annotated to the transcription start site of *SLA2*, while *FAM83C* and *EIF6* are positioned downstream within the same locus, supporting potential *cis*-regulatory relationships. For the blood eQTM, the RNA gene *LOC105372599* is located at an approximately 1.94 Mb distance from cg00036588, downstream in genomic coordinates but upstream relative to transcription on the minus strand. No eQTMs reached an FDR <0.20 for cg16759041.

## Discussion

This genome-wide discovery analysis for the role of DNA methylation in type 2 diabetes in West Africans identified 57 CpG sites associated with type 2 diabetes development and related glycaemic traits. MR analyses provided suggestive evidence for two of these CpG sites as causal for type 2 diabetes. The causal associations were further supported by longitudinal effects of these CpGs on HbA_1c_ levels. Additionally, one of the causal CpG sites showed a correlation with the expression of two genes in skeletal muscle, highlighting potential functional relevance.

Inferring causality in EWAS findings is critical for distinguishing associations that reflect underlying disease mechanisms from those that are merely correlational, affected by confounding or by reverse causation. The need for applying causal inference methods to type 2 diabetes EWAS was emphasised in a recent review [10]. To date, EWAS MR studies on type 2 diabetes have predominantly used data from European-ancestry populations [32–34]. Given the large influence of both genetic and environmental factors on DNA methylation, it is likely that CpGs exhibit population-specific effects, emphasising the need for inclusion of diverse populations [35]. Our study represents the first application of MR to identify CpGs causal for type 2 diabetes in a non-European population. MR strengthens causal claims for specific CpGs when MR assumptions are met, namely the relevance, independence and exclusion restriction assumptions. It is never possible to prove definitively that these assumptions hold; thus, additional evidence is desirable to support claims of causality [36]. In our study, we provided this through longitudinal associations and correlations with gene expression in relevant tissues, further reinforcing the causal claims.

The cg00036588 CpG site, for which we found evidence of causality using meQTLs in blood, has not previously been reported in any EWAS [37], making our EWAS in Ghanaians the first to detect it. Its relative stability over time compared with its EWAS effect size suggests a potential utility of cg00036588 as a biomarker. Larger longitudinal studies are needed to evaluate its predictive value for the incidence of type 2 diabetes and its utility in the early detection of type 2 diabetes. Our eQTM analyses revealed that while this CpG site is the transcription start site of *SLA2*, it was correlated with expression levels of *FAM83C* in skeletal muscle and with the RNA gene *LOC105372599* in blood. *FAM83C* and *LOC105372599* have no known function or reported association in relation to cardiometabolic traits. At a relaxed significance threshold, this CpG was also correlated with *EIF6*. *EIF6* codes for eIF6 protein, which has been proposed as a therapeutic target for type 2 diabetes, as well as for obesity and cancer [38]. In addition, the eIF6 protein has been implicated in the pathogenesis of diabetic ulcers [39], and eIF6 knockdown in hepatocytes affected insulin response [40]. Additionally, this CpG was found to be differentially methylated between young and older muscle stem cells, reinforcing its potential role in muscle tissue [41]. However, functional analyses are required to establish whether the effects of this CpG on *EIF6* in muscle, identified using a small sample size and exploratory threshold, represent a pathway suitable for therapeutic targeting.

The CpG site cg16759041 was negatively associated with HbA_1c_ in cross-sectional EWAS analyses using DNA methylation data profiled from white blood cells, yet was positively associated with type 2 diabetes in MR leveraging meQTLs identified in hepatocytes. A positive association was also found with HbA_1c_ in longitudinal analyses. A comparison of CpGs measured in paired liver and blood samples showed that tissue correlation varied widely across CpGs [42]. This may suggest that while the cross-sectional EWAS identified cg16759041 as relevant, the negative association in blood may reflect reverse causation or an immune cell-specific effect, whereas the positive associations observed in hepatocyte MR and longitudinal analyses point to a potential causal role for cg16759041 in type 2 diabetes pathophysiology. Little is known about the biological significance of this CpG site or the biological mechanisms by which it may affect type 2 diabetes. This CpG site has not been reported previously in relation to any trait in the EWAS Atlas. While the probe is included on the EPIC array, it was not present on the earlier 450k array, which was used for the majority of EWAS published to date [37]. In addition, the EWAS Atlas is dominated by European-ancestry EWAS. cg16759041 is annotated to an intergenic region and the nearest upstream and downstream genes (*MDD* and *SMIM36*) have not been implicated in glycaemic traits. In addition, the comparison of our HbA_1c_ EWAS effect size with the change in methylation over a period of nearly 7 years for this CpG showed a similar magnitude, suggesting that time-related variation may obscure disease-associated variation for this CpG. Further investigation is needed using multi-tissue approaches to disentangle the potential role of this CpG in type 2 diabetes pathophysiology.

Our findings are consistent with other studies that did not find evidence for causality for most CpGs cross-sectionally associated with cardiometabolic diseases. Two studies conducted in predominantly European-ancestry populations each identified one CpG with evidence for a causal effect on type 2 diabetes. Specifically, CpG cg00574958 (*CPT1A*) was identified from 16 CpGs tested in a British cohort [32], and cg25536676 (*DHCR24*) was identified from 30 CpGs tested in the largest meta-EWAS of type 2 diabetes in Europeans [33]. While we tested cg00574958 for causality in our study, cg25536676 was not associated with type 2 diabetes or related traits in EWAS among West Africans. Similarly, a lack of causal CpGs has been reported for other cardiometabolic traits. For example, MR analysis on BMI-associated CpGs using data from the Framing Heart Study identified only two nominally significant causal associations with BMI [43]. These findings suggest that the majority of CpGs identified in EWAS are unlikely to have a direct causal role. However, those few with causal evidence hold promise as biomarkers or therapeutic targets.

The meQTLs identified using DNA methylation data from hepatocytes differed from those identified in blood. Previous studies have shown that tissue specificity for meQTLs is common. Notably, meQTL mapping using DNA methylation data on nine tissues from 424 GTEx samples found that only 16% of the meQTLs identified had a secondary signal in at least one other tissue [44]. Our findings suggest that tissue specificity matters in type 2 diabetes pathophysiology. However, alternative explanations must be considered as well. Differences in genetic ancestry, the presence of European admixture in African Americans (unlike the West Africans in our study), gene–environment interactions and differences in statistical power due to the substantially smaller sample size of the hepatocyte dataset may have limited the detection of shared meQTLs. Larger, harmonised multi-tissue meQTL datasets in diverse populations are needed to determine the role of tissue-specific epigenetic effects in type 2 diabetes pathophysiology.

The strengths of this study include the use of two independent cohorts of West Africans, an understudied population in epigenetic research. Additionally, the inclusion of hepatocytes alongside white blood cells, the most studied tissue in EWAS, allowed us to assess tissue-specific effects, providing deeper insights into type 2 diabetes pathophysiology. Finally, our study used some of the limited longitudinal data sources available in sub-Saharan Africa, and analyses of these data further strengthened our causal claims. However, our study is not without limitations. The sample size is relatively small compared with studies in other populations, which has reduced the statistical power to detect associations with small effect size. The limited sample size also precluded sex-stratified analyses, although sex differences in DNA methylation patterns and in the pathophysiology of type 2 diabetes have been reported. Given that approximately 50–70% of participants across the different analytic subsets were women, our findings are broadly applicable to both sexes. However, we cannot exclude the possibility of sex-specific associations. While we found evidence for cg00036588 and cg16759041 being causally linked to type 2 diabetes, we cannot exclude the possibility that other identified CpGs may also have causal roles. In particular, for CpGs with smaller estimated causal effects, our study had limited power to detect significant associations. In addition, meQTL instruments were estimated in the AADM study, a type 2 diabetes case–control cohort, which may introduce ascertainment-related bias when both the genetic instrument and the CpG site are independently associated with type 2 diabetes. However, EWAS of type 2 diabetes were conducted exclusively in the population-based RODAM cohort, EWAS of glycaemic traits in the AADM study were effectively restricted to control participants and the use of *cis* meQTL instruments reduces the likelihood that instruments are disease associated. Further studies in population-based cohorts with larger sample size are needed to more definitively assess causality for these sites. Our causal estimates are also affected by the standard MR limitations related to its reliance on a few assumptions. We minimised the likelihood of confounding by excluding instruments that had a nominal association with potential confounders in our dataset, by using a two-sample approach and by testing for heterogeneity following the Rucker framework. Furthermore, our causal findings were supported by longitudinal analyses, while the eQTM analyses in relevant tissues supported a potential regulatory role. It should be noted that, because our study focused on individuals with untreated type 2 diabetes, and glucose-lowering medication is known to strongly influence the epigenome, the methylation markers identified cannot be generalised to individuals receiving such treatment. Instead, they should be interpreted specifically in the context of disease onset and early development. Finally, caution should be exercised when extrapolating our findings to other African-ancestry populations due to the high genetic and environmental diversity across African populations. This diversity underscores the importance of conducting similar studies in other African populations both within Africa and in the diaspora.

### Conclusions

Our findings provide evidence for a causal role of two DNA methylation markers, cg00036588 and cg16759041, in type 2 diabetes development among West Africans. Differences observed between white blood cells and hepatocytes may reflect tissue specificity in causal associations (among several potential explanations), but further studies are needed to clarify the role of tissue-specific epigenetic processes in type 2 diabetes. Our findings may help advance understanding of disease mechanisms pertaining to type 2 diabetes onset and development. If validated in larger studies and supported by functional evidence, the DNA methylation markers identified could be prioritised as potential biomarkers for early detection of disease or as drug development targets.

## Supplementary Information

Below is the link to the electronic supplementary material.

ESM Figure (PDF 337 KB)

ESM Tables (XLSX 87 KB)

## Data Availability

The datasets analysed during the current study are not publicly available due to the informed consent obtained not granting permission for deposition in an open-access repository of research data. Qualified investigators may request access to the data as part of a collaboration, in accordance with the project’s institutional review board approval and the participants’ signed informed consent. Requests can be directed to the Principal Investigator of the AADM study, Dr Charles Rotimi (rotimic@mail.nih.gov) and/or the Principal Investigator of the RODAM study, Professor Charles Agyemang (c.o.agyemang@amsterdamumc.nl).

## Abbreviations

AADM: Africa America Diabetes Mellitus
eQTM: expression quantitative methylation
EWAS: Epigenome-wide association study(ies)
FDR: False discovery rate
GEO: Gene Expression Omnibus
GWAS: Genome-wide association study
HOMA: Homeostatic model assessment
HOMA-B: HOMA estimates of beta cell function
HOMA-S: HOMA estimates of insulin sensitivity
IVW: Inverse variance weighted
LD: Linkage disequilibrium
meQTLs: Methylation quantitative trait loci
MR: Mendelian randomisation
Q–Q: Quantile–quantile
RODAM: Research on Obesity and Diabetes among African Migrants

## Code availability

The analysis code is available at https://github.com/NHGRI/CRGGH/tree/meeks_MR_DNAm_T2D.
