# Supplementary material for: Causal relationship between epigenetic markers and type 2 diabetes in West African populations: a Mendelian randomisation analysis
Source: Diabetologia. 2026 Apr 24;69(7):1882–97. doi: 10.1007/s00125-026-06716-3 (PMC13236806; doi:10.1007/s00125-026-06716-3)
Supplement: Supplementary file 1 — ESM Figure (PDF 337 KB) [file 125_2026_6716_MOESM1_ESM.pdf]

ELECTRONIC SUPPLEMENTARY MATERIAL (ESM)

For manuscript entitled:
Causal relationship between epigenetic markers and type 2 diabetes in West Africans: a Mendelian randomisation analysis

Authors:
Karlijn A.C. Meeks, Eva L. van der Linden, Amy R. Bentley, Ayo P. Doumatey, Peter Henneman, Nora Franceschini, Themistocles L. Assimes, Felix P. Chilunga, Charles F. Hayfron-Benjamin, Ellis Owusu-Dabo, Guanjie Chen, Charles Agyemang, Adebowale A. Adeyemo, Charles N. Rotimi

ESM Tables (Excel file)

ESM Table 1. CpG sites associated with type 2 diabetes in West Africans.
ESM Table 2. CpG sites associated with glycaemic traits in West Africans.
ESM Table 3. SNP-Exposure associations for the 233 blood meQTLs.
ESM Table 4. SNP-type 2 diabetes associations for the 233 blood meQTLs.
ESM Table 5. Causal estimates for the association between CpG sites in blood and type 2 diabetes using IVW fixed effect model.
ESM Table 6. Causal estimates for the association between CpG sites in blood and type 2 diabetes using IVW random effect model.
ESM Table 7. Causal estimates for the association between CpG sites in blood and type 2 diabetes using a less restrictive instrument set (IVW fixed effect model).
ESM Table 8. SNP-Exposure associations for the four hepatocyte meQTLs.
ESM Table 9. SNP-type 2 diabetes associations for the four hepatocyte meQTLs.
ESM Table 10. Causal estimates for the association between CpG sites in hepatocytes and type 2 diabetes using Wald ratio analysis.

ESM Figures (Below)

ESM Fig 1. Q-Q plot of epigenome-wide P-values for type 2 diabetes (A), HbA1c (B), HOMA-S (C), and HOMA-B (D).

ESM FIGURES

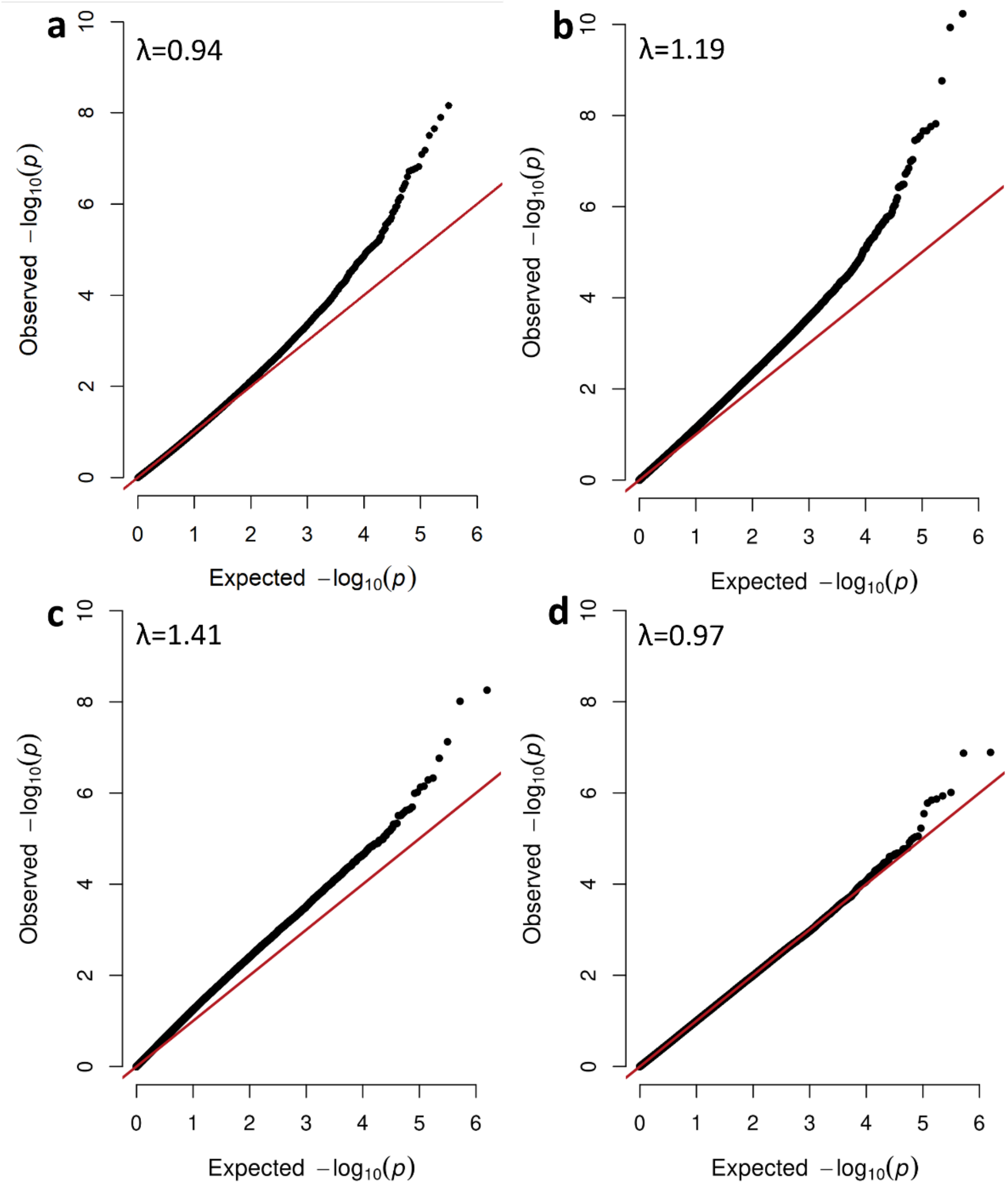

ESM Fig 1. Q-Q plot of epigenome-wide *P*-values for type 2 diabetes (a), HbA1c (b), HOMA-S (c), and HOMA-B (D).

EWAS were conducted in 879 Ghanaians for type 2 diabetes (A); 806 Ghanaians and 329 Nigerians for HbA1c (B); and 741 Ghanaians and 317 Nigerians for HOMA-S and HOMA-B (C, D). λ denotes the inflation factor.
